# Awareness of Abortion Legality and Safe Abortion Uptake in Ghana: Assessing the Moderating Role of Education in a Cross‐Sectional Study

**DOI:** 10.1002/hsr2.72928

**Published:** 2026-07-30

**Authors:** Emmanuel Banchani, Oluwaseyi Dolapo Somefun, Hengliwe Gwebu, Ololade Julius Baruwa

**Affiliations:** ^1^ Department of Global and International Health University for Development Studies Tamale Ghana; ^2^ School of Public Health University of the Western Cape Cape Town South Africa; ^3^ Department of Nursing and Public Health University of Fort Hare Alice, East London South Africa

**Keywords:** education, Ghana, legal awareness, reproductive health, safe abortion, unsafe abortion

## Abstract

**Background and Aims:**

Although awareness of abortion legality has been associated with reproductive health service utilization, little is known about whether legal awareness influences safe abortion uptake in Ghana or whether educational attainment modifies this relationship. This study examined the association between awareness of abortion legality and safe abortion uptake and assessed the moderating role of education among women in Ghana.

**Methods:**

Data were drawn from 1282 women aged 15–49 years who reported an induced abortion in the 5 years preceding the 2017 Ghana Maternal Health Survey. Abortions were classified as safe if they involved a recommended abortion method, were performed by a trained provider, and occurred in a recognized health facility. Awareness of abortion legality was assessed by asking respondents whether they knew abortion was legally permitted in Ghana under specific circumstances. Responses were categorized as aware or unaware. Covariates were selected based on existing literature relevant to abortion care‐seeking behavior. Multivariable logistic regression models were used to estimate adjusted associations between legal awareness, education, and safe abortion uptake.

**Results:**

Overall, 39.39% of abortions were classified as safe, while only 12.09% of women were aware of Ghana's abortion law. Legal awareness was strongly associated with safe abortion uptake (aOR = 2.09; 95% CI: 1.42–3.08; *p* < 0.001). Educational attainment was also positively associated with safety, with women with secondary (aOR = 1.98; 95% CI: 1.22–3.22; *p* = 0.006) and higher education (aOR = 3.06; 95% CI: 1.57–5.96; *p* = 0.001) more likely to obtain safe abortions compared with those with no education. However, education did not significantly moderate the association between legal awareness and abortion safety. Predicted probabilities indicated the highest likelihood of safe abortion among legally aware women with higher education (69%).

**Conclusion:**

These findings highlight the importance of improving women's awareness of abortion legality and strengthening women's educational attainment to enhance reproductive health autonomy and reduce unsafe abortion in Ghana.

## 1 Introduction

Globally, about 56 million induced abortions occur annually, of which approximately 45% are unsafe, making abortion a major public health concern and preventable cause of maternal morbidity and mortality, particularly in low‐ and middle‐income countries (LMICs) [1, 2]. Women's decisions to terminate pregnancies are influenced by diverse factors, including financial constraints, educational aspirations, relationship circumstances, concerns about maternal or fetal health, and family size preferences [3, 4, 5, 6]. Although induced abortion occurs worldwide, nearly 98% of unsafe abortions take place in LMICs, where restrictive social environments, limited access to quality reproductive healthcare, and structural barriers continue to compromise abortion safety [7, 8, 9, 10].

In sub‐Saharan Africa, abortion remains highly stigmatized because of prevailing religious, cultural, and social norms [11]. Beyond restrictive social attitudes, women often face inadequate access to accurate information, essential abortion medicines, trained providers, and appropriate health facilities [12, 13]. Structural barriers, including abortion‐related stigma, limited education, poor reproductive health literacy, and uncertainty regarding the legal status of abortion, may prevent women from seeking timely and safe abortion services, even where legal provisions exist [14].

Ghana represents an important example of this paradox. Although abortion has been legally permitted under specific circumstances for several decades, unsafe abortion continues to contribute substantially to maternal morbidity and mortality, accounting for approximately 10% of maternal deaths nationally [15, 16, 17, 18, 19, 20, 21]. Under Ghanaian law, abortion is permitted in cases of rape, incest, severe fetal abnormality, threats to the woman's physical or mental health, and certain circumstances involving women with mental disabilities [20, 21]. Nevertheless, abortion remains highly stigmatized, and many women continue to obtain abortions outside formal healthcare settings despite being legally eligible for safe services [18, 19].

Previous studies in Ghana have identified several determinants of safe abortion utilization, including educational attainment, socioeconomic status, place of residence, access to healthcare services, and abortion‐related stigma [17, 22, 23, 24, 25, 26, 27, 28]. However, considerably less attention has been given to women's awareness of abortion legality as a determinant of safe abortion uptake. Evidence from Ghana and other sub‐Saharan African countries suggests that knowledge of abortion laws remains limited, even in settings where abortion is legally permitted under specific conditions [22, 23]. Consequently, women who are legally eligible for safe abortion services may continue to seek unsafe procedures because they are unaware of their legal rights or where safe services can be obtained.

Educational attainment may further influence women's ability to benefit from legal awareness. Women with higher levels of education generally possess greater health literacy, reproductive autonomy, and capacity to navigate healthcare systems, potentially facilitating access to safe abortion services [23, 25]. However, education and legal awareness may influence abortion care‐seeking through different mechanisms. While education may enhance women's ability to understand health information and utilize healthcare services, awareness of abortion legality may directly influence perceptions of eligibility and willingness to seek formal care. Whether education strengthens the association between legal awareness and safe abortion uptake remains unclear.

Although previous studies have examined the prevalence, determinants, and consequences of unsafe abortion in Ghana, no nationally representative study has investigated whether awareness of abortion legality is associated with safe abortion uptake or whether this relationship differs according to women's educational attainment [17, 23, 25, 26]. Addressing this evidence gap is important because improving women's knowledge of abortion laws may represent a practical and scalable strategy for reducing unsafe abortion and improving reproductive health outcomes. Therefore, this study examined the association between awareness of abortion legality and safe abortion uptake among women in Ghana and assessed whether educational attainment moderates this relationship. We hypothesized that women who were aware of the legal conditions under which abortion is permitted would be more likely to obtain safe abortion services and that this association would be stronger among women with higher educational attainment.

## 2 Methods

### 2.1 Study Design and Data Source

This study is a secondary data analysis of data from the 2017 Ghana Maternal Health Survey (GMHS), a nationally representative, cross‐sectional survey that collected detailed information on women's reproductive health, maternal health service utilization, and abortion‐related experiences. The GMHS was implemented by the Ghana Statistical Service (GSS) in collaboration with the Ghana Health Service (GHS), with technical support from ICF International under the Demographic and Health Surveys (DHS) Program, from June to October 2017 [29].

The GMHS employed a two‐stage stratified cluster sampling design. In the first stage, 900 enumeration areas (EAs) were selected from the 2010 Ghana Population and Housing Census sampling frame (466 urban and 434 rural). In the second stage, a fixed number of 30 households per EA were randomly selected using systematic sampling. All women aged 15–49 years who were usual residents or slept in the selected households the night before the survey were eligible to participate. The GMHS women's questionnaire contained modules on fertility history, maternal health, and abortion experiences, including details on methods used, providers, and places of abortion, allowing for the construction of a measure of abortion safety consistent with WHO's guideline [30]. Standardized questionnaires and data collection procedures developed by the GSS and its partners were used throughout the survey. Survey instruments were extensively pretested, interviewer training was standardized, and rigorous quality‐control procedures were implemented during data collection to ensure the reliability and validity of the information collected.

### 2.2 Study Population and Sample Size

**Figure 1:**
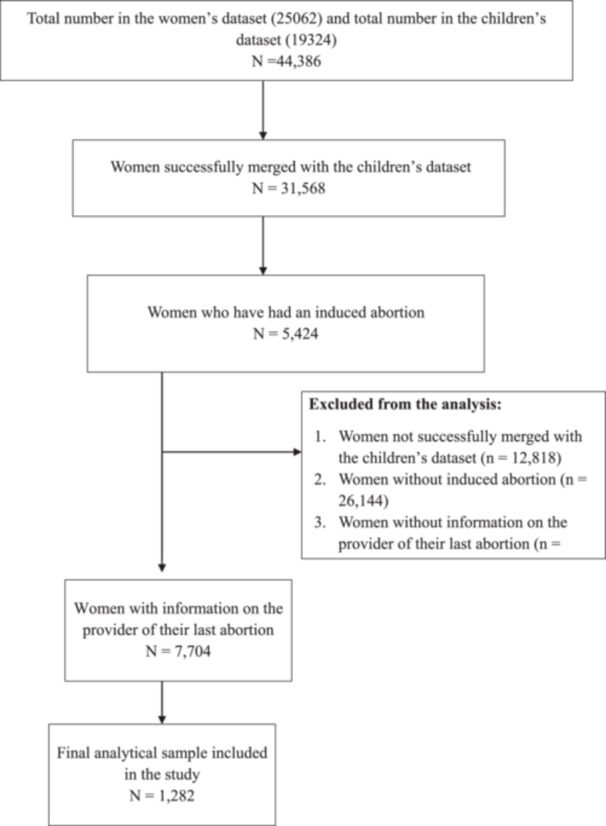
Sample selection flow diagram.

This analysis focused on women aged 15–49 years who reported where and by whom an induced abortion was performed during the 5‐year reference period preceding the survey. Of the women interviewed, 1282 were eligible for inclusion in our analysis. These women had experienced an induced abortion within the 5 years preceding the survey and completed the abortion module containing detailed follow‐up questions on abortion. Respondents with missing data on the outcome variable (safe abortion) or key explanatory variables were excluded. Figure 1 provides the participant selection process. Two GMHS data sets were merged to create the analytic data set: the individual women's data set (IR file), which contains information on respondents' sociodemographic and reproductive characteristics, as well as their awareness of abortion legality; and the child data set (KR file), which contains detailed information on pregnancies, abortion histories, providers, and places where abortions were obtained.

### 2.3 Outcome Variable

The outcome variable, *safe abortion*, was defined in accordance with the World Health Organization (WHO) abortion safety framework, which classifies abortions based on three dimensions: abortion method, provider, and place of service. Following WHO recommendations, abortions were classified as safe when all three criteria were met: (1) use of a recommended medical or surgical abortion method, (2) provision by a medically trained provider, and (3) performance in a recognized health facility [30]. Abortions that did not meet one or more of these criteria were classified as unsafe.

### 2.4 Main Independent Variable

The key exposure variable was *awareness of abortion legality*. Women were asked a single question “Is abortion legal in Ghana?" Responses were categorized as “aware” [1] and “unaware” (0).

### 2.5 Covariates

Based on previous literature and their relevance to abortion care utilization, several sociodemographic and reproductive variables were included as covariates in this study [31, 32, 33]. *Age* was recorded in categories according to the respondent's age at the time of the survey and measured in our study (15–24, 25–34, and 35–49 years). *Marital status* was measured as a binary variable indicating whether the respondent had ever been married (currently married, cohabitating, separated, divorced, and widowed) or had never been married at the time of the survey. *Education* level was defined as the highest level of formal education attained and was categorized as no education, primary, secondary, or higher education.

The household *wealth index* was constructed by the DHS using Principal Component Analysis based on household assets such as ownership of a television, car, livestock, land, and bank account; housing characteristics such as the type of floor, roof, and walls; and access to basic utilities such as electricity and drinking water. From the data set, the household wealth index was categorized into five categories, ranging from poorest to richest. For this analysis, the wealth index was recoded into three categories: poor, middle, and rich. *Religion* was categorized as Catholic, Christian, Muslim, or other religions. *Ethnicity* was grouped as Akan, Ga/Dangme, Ewe, Mole‐Dagbani, and other ethnicities, following the classification used by Wongnaah et al. [33]. *Region* of residence was classified as Western, Central, Greater Accra, Volta, Eastern, Ashanti, Brong Ahafo, Northern, Upper East, and Upper West, while place of residence was dichotomized as urban or rural.

The *reason for abortion* was categorized using previous guidelines from Chae et al.'s [32] study into health‐related, personal, social, economic, and other reasons. Health‐related reasons included concerns about the mother's health, risk of birth defects, or a nonviable pregnancy. Personal reasons comprised being too young to have a child, not ready for motherhood, wanting to delay or space childbearing, or desiring no more children. Social reasons encompassed not wanting to remain with the father, denial of paternity, death of the father, rape, or avoiding social stigma. Economic reasons referred to financial constraints, lack of childcare support, or the desire to continue schooling or employment. Other reasons included parental pressure and other unspecified motivations.

Additional reproductive and behavioral variables were also included. *Parity* was defined as the total number of children ever born and categorized as none, 1–2 births, or 3+ births. *Contraceptive use* was defined as the use of any method to prevent pregnancy and classified as none, modern, or traditional methods. *Early sexual debut* was defined as having first sexual intercourse before the age of 15 years, following [34, 35], and measured as a binary variable (yes/no). *Media exposure* was assessed based on whether respondents reported reading newspapers, listening to the radio, or watching television at least once per week. Women who engaged in any of these media activities weekly were classified as exposed to media, whereas those who did not were considered not exposed.

### 2.6 Statistical Analysis

This study analyzed data from 1282 women who reported an induced abortion during the past 5 years preceding the survey. A four‐step analytical strategy was employed to examine the relationship between awareness of abortion legality and safe abortion uptake. First, descriptive statistics were computed to summarize the prevalence of safe abortion and the distribution of all explanatory variables across the sample. Second, multivariable logistic regression analyses were fitted to estimate the adjusted associations between awareness of abortion legality and safe abortion. Two models were specified. Model 1 estimated the unadjusted (bivariate) association, while Model 2 adjusted for sociodemographic and reproductive characteristics. Results were presented as adjusted odds ratios (aORs) with corresponding 95% confidence intervals (CIs).

Before fitting the regression analyses, multicollinearity was assessed using the Variance Inflation Factor (VIF). No evidence of problematic multicollinearity was observed, with VIF < 3 (Table S1). In addition, model fit was assessed using the Hosmer–Lemeshow goodness‐of‐fit test and Nagelkerke *R* ^2^ statistics [36, 37]. The final model demonstrated acceptable fit according to the Hosmer–Lemeshow goodness‐of‐fit test (*p* > 0.05). In addition, the model demonstrated good explanatory power with a Nagelkerke *R* ^2^ of 0.391, indicating that approximately 39.1% of the variation in safe abortion uptake was explained by the variables included in the model.

Third, a moderation analysis was conducted to test whether education level modified the relationship between awareness of abortion legality and safe abortion. Moderation was assessed by introducing a multiplicative interaction term between awareness of abortion legality and educational attainment in the fully adjusted model. Finally, predicted probabilities and marginal effects were then computed to visualize the interaction, highlighting differences in safe abortion uptake by education level and legal awareness status. Predicted probabilities were estimated using post‐estimation margins procedures following logistic regression.

All analyses were conducted using Stata version 16 (StataCorp, College Station, TX, USA). The data were weighted using the GMHS sampling weights to account for the complex survey design, stratification, and clustering, ensuring nationally representative estimates. Statistical significance was determined at *p* < 0.05.

### 2.7 Ethical Considerations

The GMHS protocol received ethical approval from the Ghana Health Service Ethics Review Committee and the ICF Institutional Review Board (IRB). This study used anonymized, publicly available secondary data from the DHS Program and therefore did not require additional ethical clearance. Written informed consent was obtained from all participants during the survey.

## 3 Results

### 3.1 Sample Selection

The sample selection process is presented in Figure 1. The study utilized data from both the women's data set (*n* = 25,062) and the children's data set (*n* = 19,324), yielding a combined sample of 44,386 records. Following data set linkage, 31,568 women were successfully merged with the children's data set and were eligible for further assessment. Among these women, 5424 reported having had an induced abortion. After applying the exclusion criteria, 1282 women with complete information on the provider of their last abortion remained eligible for analysis. Therefore, the final analytical sample comprised 1282 women who had experienced an induced abortion and had complete information on the provider and place of abortion care.

### 3.2 Characteristics of the Study Population

Table 1 presents the characteristics of women who reported an induced abortion in the 2017 GMHS (*N* = 1282). Overall, 39.39% of abortions were classified as safe, while 12.09% of respondents were aware of the legal status of abortion in Ghana.

**Table 1:** Characteristics of the study population.

Variables	Sample size (*n*/*N*)	Percentage (95% CI)
Outcome		
Safe abortion	505/1282	39.39 (36.75–42.10)
Main independent variable		
Awareness of abortion legality	155/1282	12.09 (10.41–13.99)
Covariates		
Age		
15–24	515/1282	40.17 (37.52–42.88)
25–34	558/1282	43.53 (40.83–46.26)
35–49	209/1282	16.30 (14.38–18.43)
Education		
No education	136/1282	10.61 (9.04–12.42)
Primary	213/1282	16.61 (14.68–18.75)
Secondary	841/1282	65.60 (62.96–68.15)
Higher	92/1282	7.18 (5.89–8.72)
Ever married	871/1282	67.94 (65.33–70.44)
Household wealth index		
Poor	335/1282	26.13 (23.80–28.61)
Middle	305/1282	23.79 (21.54–26.20)
Rich	642/1282	50.08 (47.34–52.82)
Religion		
Catholic	138/1282	10.76 (9.18–12.58)
Christians	976/1282	76.13 (73.72–78.39)
Muslims	137/1282	10.69 (9.11–12.50)
Others	31/1282	2.42 (1.70–3.42)
Rural residence	450/1282	35.10 (32.53–37.76)
Ethnicity		
Akan	707/1282	55.15 (52.41–57.86)
Ga/Dangme	90/1282	7.02 (5.74–8.56)
Ewe	163/1282	12.71 (11.00–14.66)
Mole‐Dagbani	185/1282	14.43 (12.61–16.47)
Others	137/1282	10.69 (9.11–12.50)
Region		
Western	206/1282	16.07 (14.16–18.18)
Central	97/1282	7.57 (6.24–9.15)
Greater Accra	194/1282	15.13 (13.27–17.20)
Volta	89/1282	6.94 (5.67–8.47)
Eastern	121/1282	9.44 (7.95–11.17)
Ashanti	246/1282	19.19 (17.12–21.44)
Brong Ahafo	168/1282	13.10 (11.36–15.07)
Northern	48/1282	3.74 (2.83–4.94)
Upper East	40/1282	3.12 (2.30–4.23)
Upper West	73/1282	5.69 (4.55–7.11)
Media exposure	493/1282	38.46 (35.83–41.15)
Early sexual debut	202/1282	15.76 (13.86–17.86)
Parity		
None	420/1282	32.76 (30.24–35.38)
1–2 births	538/1282	41.97 (39.29–44.69)
3+	324/1282	25.27 (22.97–27.73)
Contraceptive use		
No method	757/1282	59.05 (56.33–61.71)
Modern methods	432/1282	33.70 (31.16–36.34)
Traditional methods	93/1282	7.25 (5.95–8.81)
Reason for abortion		
Health‐related	104/1282	8.11 (6.74–9.74)
Personal	462/1282	36.04 (33.45–38.71)
Social	218/1282	17.00 (15.04–19.16)
Economic	406/1282	31.67 (29.18–34.27)
Others	92/1282	7.18 (5.88–8.73)

The majority of women were aged 25–34 years (43.53%), followed by those aged 15–24 years (40.17%). Most respondents had attained secondary education (65.6%), while 10.61% had no formal education and 7.18% had attained higher education. Approximately 67.94% had ever been married, and 26.13% belonged to poor households. By religion, 76.13% identified as Christian and 10.69% as Muslim. The Akan ethnic group constituted the largest proportion of respondents (55.15%), followed by Mole‐Dagbani (14.43%) and Ewe (12.71%). Regionally, most participants were from Ashanti (19.19%), Western (16.07%), and Greater Accra (15.13%) regions.

In terms of behavioral and reproductive characteristics, 38.46% reported exposure to media, and 15.76% had an early sexual debut before age 15. About 41.97% had one or two live births, 32.76% had none, and 25.27% had three or more births. Regarding contraceptive use, 59.05% used no method, 33.7% used modern methods, and 7.25% used traditional methods. Regarding the main reasons for abortion, personal factors accounted for the largest share (36.04%), followed by economic reasons (31.67%), social factors (17%), while health‐related reasons accounted for 8.11%.

### 3.3 Multivariable Analysis of Factors Associated With Safe Abortion Uptake

Results from the multivariable logistic regression analysis (Table 2) showed that awareness of abortion legality was a strong and consistent predictor of safe abortion uptake. Women who were aware that abortion is legal in Ghana were more than twice as likely to have had a safe abortion compared with those who were unaware (aOR = 2.09; 95% CI: 1.42–3.08; *p* < 0.001).

**Table 2:** Multivariable logistic regression analysis showing the association between awareness of abortion legality and safe abortion uptake in Ghana.

	Model 1 (unadjusted OR)		Model 2 (adjusted OR)	
	uOR (95% CI)	*p*	aOR (95% CI)	*p*
Awareness of abortion legality	2.77 (1.96–3.91)	< 0.001	2.09 (1.42–3.08)	< 0.001
Age				
15–24	1		1	
25–34	1.56 (1.21–2.00)	0.001	1.40 (1.02–1.93)	0.040
35–49	2.20 (1.59–3.06)	< 0.001	1.65 (1.01–2.69)	0.046
Education				
No education	1		1	
Primary	0.90 (0.57–1.41)	0.635	1.48 (0.88–2.50)	0.141
Secondary	1.10 (0.76–1.60)	0.620	1.98 (1.22–3.22)	0.006
Higher	2.44 (1.42–4.21)	0.001	3.06 (1.57–5.96)	0.001
Ever married	1.33 (1.04–1.70)	0.021	1.07 (0.76–1.49)	0.702
Household wealth index				
Poor	1		1	
Middle	0.91 (0.66–1.25)	0.558	1.12 (0.77–1.62)	0.547
Rich	1.24 (0.95–1.63)	0.116	1.32 (0.92–1.91)	0.134
Religion				
Catholic	1		1	
Christians	0.62 (0.44–0.89)	0.010	0.65 (0.44–0.97)	0.035
Muslims	0.80 (0.50–1.29)	0.363	0.71 (0.42–1.20)	0.205
Others	0.42 (0.18–0.98)	0.045	0.41 (0.18–0.95)	0.038
Rural residence	1.04 (0.82–1.32)	0.743	1.19 (0.88–1.60)	0.262
Ethnicity				
Akan	1		1	
Ga/Dangme	0.69 (0.42–1.11)	0.125	0.61 (0.34–1.08)	0.090
Ewe	1.55 (1.10–2.18)	0.013	1.15 (0.73–1.84)	0.543
Mole‐Dagbani	1.31 (0.95–1.83)	0.102	0.90 (0.54–1.50)	0.672
Others	1.20 (0.83–1.75)	0.331	0.96 (0.60–1.54)	0.854
Region				
Western	1		1	
Central	0.84 (0.51–1.40)	0.509	0.78 (0.45–1.36)	0.386
Greater Accra	1.15 (0.77–1.72)	0.497	1.05 (0.64–1.74)	0.838
Volta	1.60 (0.97–2.65)	0.068	1.43 (0.75–2.70)	0.275
Eastern	1.47 (0.93–2.33)	0.096	1.45 (0.87–2.39)	0.152
Ashanti	0.81 (0.55–1.20)	0.287	0.77 (0.51–1.18)	0.233
Brong Ahafo	0.90 (0.59–1.38)	0.635	0.97 (0.62–1.52)	0.904
Northern	2.20 (1.16–4.16)	0.015	2.85 (1.38–5.91)	0.005
Upper East	1.89 (0.95–3.74)	0.68	1.55 (0.66–3.64)	0.313
Upper West	1.58 (0.92–2.70)	0.099	1.59 (0.81–3.12)	0.179
Media exposure	0.97 (0.77–1.22)	0.796	0.97 (0.75–1.26)	0.811
Early sexual debut	1.01 (0.74–1.37)	0.946	1.28 (0.91–1.80)	0.158
Parity				
None	1		1	
1–2 births	0.99 (0.76–1.29)	0.927	0.99 (0.70–1.39)	0.944
3+	1.53 (1.14–2.05)	0.005	1.34 (0.83–2.16)	0.228
Contraceptive use				
No method	1		1	
Modern methods	1.02 (0.80–1.30)	0.879	1.06 (0.81–1.38)	0.665
Traditional methods	0.93 (0.60–1.45)	0.747	1.14 (0.72–1.82)	0.573
Reason for abortion				
Health‐related	1		1	
Personal	0.11 (0.07–0.20)	< 0.001	0.13 (0.07–0.23)	< 0.001
Social	0.12 (0.07–0.22)	< 0.001	0.14 (0.08‐0.27)	< 0.001
Economic	0.11 (0.06–0.18)	< 0.001	0.12 (0.07–0.23)	< 0.001
Others	0.13 (0.07–0.26)	< 0.001	0.18 (0.09–0.37)	< 0.001

Age was also significantly associated with abortion safety. Compared with adolescents and young women aged 15–24 years, women aged 25–34 years (aOR = 1.40; 95% CI: 1.02–1.93; *p* = 0.040) and 35–49 years (aOR = 1.65; 95% CI: 1.01–2.69; *p* = 0.046) had higher odds of having a safe abortion. Women with secondary (aOR = 1.98; 95% CI: 1.22–3.22; *p* = 0.006) and higher education (aOR = 3.06; 95% CI: 1.57–5.96; *p* = 0.001) were significantly more likely to undergo safe abortion compared to those with no formal education.

Religion was also associated with safe abortion uptake; Catholic women were more likely to report safe abortions compared to women of other faiths, with lower odds observed among Christian (aOR = 0.65; 95% CI: 0.44–0.97; *p* = 0.035) and other religious groups (aOR = 0.41; 95% CI: 0.18–0.95; *p* = 0.038). Women residing in the Northern Region had higher odds of safe abortion (aOR = 2.85; 95% CI: 1.38–5.91; *p* = 0.005) compared with those in the Western Region. The reasons for abortion were strongly associated with abortion safety. Women who cited personal (aOR = 0.13; 95% CI: 0.07–0.23; *p* < 0.001), social (aOR = 0.14; 95% CI: 0.08–0.27; *p* < 0.001), economic (aOR = 0.12; 95% CI: 0.07–0.23; *p* < 0.001), or other (aOR = 0.18; 95% CI: 0.09–0.37; *p* < 0.001) reasons had significantly lower odds of safe abortion compared to those who terminated pregnancy for health‐related reasons.

### 3.4 Moderation Analysis of Awareness of Abortion Legality and Education on Safe Abortion Uptake

Results from the interaction model (Table 3) indicated that education did not significantly moderate the association between awareness of abortion legality and the uptake of safe abortion in Ghana. Although the direction of association suggested that the effect of legal awareness on safe abortion increased with education level, the interaction terms comparing women with primary education (aOR = 0.23, 95% CI = 0.04–1.12, *p* = 0.069), secondary education (aOR = 2.34, 95% CI = 0.64–8.59, *p* = 0.200) and higher education (aOR = 1.82, 95% CI = 0.40–8.27, *p* = 0.435) did not reach statistical significance.

**Table 3:** Logistic regression model for safe abortion uptake with interaction effects for awareness of abortion legality and education, adjusting for covariates.

	aOR (95% CI)	*p*
Awareness of abortion legality	1.23 (0.37–4.10)	0.731
Awareness of abortion legality##Education		
No awareness## no education	1	
Awareness##Primary	0.23 (0.04–1.12)	0.069
Awareness##Secondary	2.34 (0.64–8.59)	0.200
Awareness##Higher	1.82 (0.40–8.27)	0.435
Age		
15–24	1	
25–34	1.41 (1.02–1.95)	0.037
35–49	1.70 (1.04–2.78)	0.035
Education		
No education	1	
Primary	1.61 (0.94–2.75)	0.084
Secondary	1.86 (1.13–3.06)	0.014
Higher	2.90 (1.36–6.19)	0.006
Ever married	1.07 (0.77–1.51)	0.676
Household wealth index		
Poor	1	
Middle	1.13 (0.78–1.64)	0.512
Rich	1.32 (0.92–1.91)	0.135
Religion		
Catholic	1	
Christians	0.65 (0.43–0.97)	0.035
Muslims	0.71 (0.42–1.21)	0.207
Others	0.46 (0.20–1.03)	0.060
Rural residence	1.19 (0.88–1.61)	0.252
Ethnicity		
Akan	1	
Ga/Dangme	0.60 (0.34–1.07)	0.082
Ewe	1.12 (0.70–1.79)	0.631
Mole‐Dagbani	0.92 (0.55–1.54)	0.751
Others	0.92 (0.57–1.50)	0.745
Region		
Western	1	
Central	0.79 (0.45–1.38)	0.404
Greater Accra	1.06 (0.64–1.76)	0.816
Volta	1.50 (0.79–2.83)	0.215
Eastern	1.43 (0.86–2.38)	0.164
Ashanti	0.76 (0.50–1.17)	0.215
Brong Ahafo	0.98 (0.62–1.53)	0.918
Northern	2.89 (1.39–5.99)	0.004
Upper East	1.52 (0.64–3.61)	0.346
Upper West	1.58 (0.80–3.10)	0.186
Media exposure	0.94 (0.73–1.23)	0.660
Early sexual debut	1.26 (0.90–1.78)	0.182
Parity		
None	1	
1–2 births	0.99 (0.70–1.40)	0.958
3+	1.34 (0.83–2.17)	0.232
Contraceptive use		
No method	1	
Modern methods	1.06 (0.81–1.38)	0.679
Traditional methods	1.15 (0.72–1.84)	0.555
Reason for abortion		
Health‐related	1	
Personal	0.12 (0.07–0.22)	< 0.001
Social	0.12 (0.07–0.25)	< 0.001
Economic	0.12 (0.06–0.21)	< 0.001
Others	0.17 (0.08–0.35)	< 0.001

**Figure 2:**
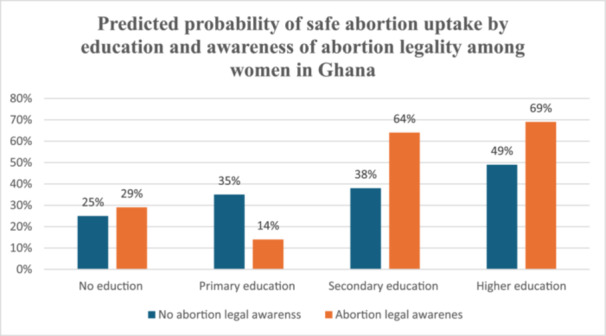
Percentage predicted probabilities of safe abortion by education and awareness of abortion legality among women in Ghana.

Nevertheless, the adjusted predicted probabilities (Figure 2) showed a descriptive pattern of higher safe abortion uptake among women with greater educational attainment and awareness of abortion legality. For example, the predicted probability of obtaining a safe abortion increased from approximately 25% among women with no education who were unaware of abortion legality to approximately 69% among women with higher education who were aware of the legal status of abortion. However, these differences should be interpreted cautiously because the interaction between education and awareness of abortion legality was not statistically significant.

## 4 Discussion

This study examined the association between women's awareness of abortion legality and the uptake of safe abortion in Ghana, and further investigated whether formal education moderates this relationship. Our analysis of a nationally representative sample of women who had an induced abortion yielded several key findings that align with existing studies.

First, we found that awareness of Ghana's abortion law was strikingly low at a national level, with only 12.09% of women who reported an induced abortion being aware that abortion is legally permitted under specific circumstances. This finding highlights a substantial gap between Ghana's legal framework and women's awareness of their legal reproductive rights in Ghana, a phenomenon that is replicated in other contexts. For instance, a national study in Zambia found that only 16% of women could correctly identify the legal grounds for abortion [38]. This pattern of limited awareness extends beyond these two countries as a systematic review synthesizing evidence from 12 countries concluded that correct knowledge of abortion law is generally poor among women, even in settings with relatively liberal laws, and noted that knowledge of legal reforms often fails to diffuse effectively into the population [39]. Despite the generally low levels of knowledge, substantial differences exist across sociodemographic groups. For instance, a study among Ghanaian undergraduate university students found that a majority (53.3%) were aware of the legal conditions under which abortion is permitted, highlighting important educational and socioeconomic disparities in abortion‐related knowledge. These findings are consistent with broader evidence showing that awareness of abortion laws often remains unevenly distributed, even in settings where abortion is legally permitted under specific circumstances. A systematic review by Assifi and colleagues found that women's knowledge of abortion laws is generally poor globally and tends to be lower among women with limited education and those living in rural areas [39]. Collectively, these findings suggest that legal reforms alone may be insufficient to improve safe abortion access unless accompanied by deliberate efforts to disseminate accurate information to women who are most vulnerable to unsafe abortion practices.

Second, the role of education was prominent and direct. We observed a clear educational gradient that aligns with previous studies, showing that education enhances women's reproductive autonomy, health literacy, and capacity to navigate healthcare systems [40, 41]. Women with secondary and higher education were about two folds and three folds times more likely, respectively, to have a safe abortion than women with no formal education. Higher educational attainment may enhance women's ability to identify qualified providers, understand legal entitlements, interpret health information, and overcome barriers to accessing formal reproductive healthcare services. Although educational attainment was independently associated with safe abortion uptake, it did not significantly modify the relationship between awareness of abortion legality and abortion safety. This suggests that the benefits of legal awareness may extend across educational groups rather than being restricted to highly educated women. While the predicted probabilities indicated that women who were both legally aware and highly educated had the greatest likelihood of obtaining a safe abortion, the interaction terms were not statistically significant. These findings suggest that education and legal awareness represent complementary, yet largely independent, pathways to safer abortion care. This finding aligns with emerging evidence suggesting that legal awareness, reproductive rights literacy, and access to safe abortion services are shaped by broader structural factors beyond formal education alone. For example, a recent study on abortion stigma among healthcare providers reported that raising awareness of legal rights and improving reproductive health education were essential for reducing stigma and facilitating equitable access to abortion services [42]. This finding suggests that educational interventions alone may not be sufficient; broader legal literacy initiatives and supportive health system environments are also needed to improve access to safe abortion care.

Third, our findings highlight the influence of the reason for seeking an abortion. Women who terminated pregnancies for health‐related reasons had significantly higher odds of a safe abortion compared to those citing personal, social, or economic reasons. This finding is consistent with Ghana's legal framework, which explicitly permits abortion on health grounds [20, 43]. It suggests that when the reason aligns closely with the legal criteria, women may be more readily channeled into the formal healthcare system by providers, or may feel more justified in seeking formal care, thereby increasing their likelihood of obtaining safe abortion services.

Other factors, such as older age (25–49 years) and residing in the Northern Region, were also associated with safer practices. The observed association with age may reflect greater autonomy, financial independence, or experience with the health system among older women. The observed regional differences may reflect disparities in health infrastructure, provider density, and the implementation of reproductive health policies across Ghana.

Our study contributes to a growing body of literature demonstrating that awareness of abortion legality is not merely a legal or informational issue but an important determinant of reproductive health behavior. Consistent with evidence from global reviews, women who understand the legal context of abortion may be more likely to seek services from qualified providers and recognized health facilities rather than resorting to informal or unsafe options [39]. Recent discussions within the reproductive rights literature further emphasize that access to accurate legal information represents a critical component of reproductive autonomy and informed decision‐making [43]. By demonstrating a positive association between legal awareness and safe abortion uptake, our findings provide empirical support for efforts to integrate abortion law education into reproductive health programs and public health communication strategies. Furthermore, by examining the moderating role of education, this study extends previous research beyond simple associations between legal awareness and reproductive health outcomes. Although education did not significantly modify the observed relationship, the findings underscore the importance of pursuing parallel strategies that simultaneously improve educational opportunities, strengthen reproductive health literacy, reduce abortion stigma, and expand access to accurate legal information.

## 5 Strengths and Limitations

This study has several strengths, including the use of a large, nationally representative data set, the application of a WHO‐based standardized definition for abortion safety, and a robust analytical approach that included moderation analysis. However, several limitations must be acknowledged. The cross‐sectional design precludes causal inference because causal relationships cannot be established. The measure of legal awareness of abortion legality was relatively broad and did not assess understanding of the specific legal conditions, which may be more relevant. A further limitation relates to the exclusion of women with missing information on the provider of their most recent abortion. If women excluded because of missing provider information differed systematically from those included, selection bias may have occurred, potentially affecting the representativeness and generalizability of the findings. As with all surveys on sensitive topics, social desirability bias and underreporting of both abortion and unsafe practices are potential concerns, which may have resulted in an overestimation of safe abortion prevalence. Finally, the relatively high Nagelkerke *R* ^2^ value (0.391) suggests that the model captured several important determinants of safe abortion uptake. Nevertheless, a proportion of the variation remained unexplained, indicating that additional social, cultural, and health‐system factors not measured in the survey may also influence abortion care‐seeking behavior.

## 6 Conclusion

This study provides nationally representative evidence that awareness of abortion legality and educational attainment are important determinants of safe abortion uptake among women in Ghana. By addressing a key gap in the literature, the study demonstrates that legal awareness is associated with greater use of safe abortion services, independent of educational attainment. Although education was positively associated with safe abortion uptake, it did not significantly moderate the relationship between legal awareness and abortion safety. These findings suggest that improving women's knowledge of abortion laws through community‐based education and legal literacy initiatives may increase awareness and promote access to safe abortion services. Furthermore, strengthening access to affordable, stigma‐free abortion services and female educational attainment may contribute to reducing unsafe abortion and its associated health consequences. Bridging the gap between Ghana's legal framework and public awareness remains essential for improving women's reproductive health and advancing maternal health outcomes.

## Author Contributions

**Emmanuel Banchani:** conceptualization, writing – original draft, writing – review and editing. **Oluwaseyi Dolapo Somefun:** conceptualization, writing – original draft, writing – review and editing. **Hengliwe Gwebu:** validation, supervision, writing – original draft, writing – review and editing. **Ololade Julius Baruwa:** conceptualization, methodology, software, data curation, formal analysis. All authors contributed to the interpretation of the findings and reviewed the final version of the manuscript. All authors have read and approved the final version of the manuscript.

## Funding

The authors have nothing to report.

## Ethics Statement

As the data set is publicly available and de‐identified, no additional ethics approval was required for this secondary analysis, in accordance with national guidelines and DHS Program policies.

## Consent

As the data set is publicly available and de‐identified, no consent was required for this secondary analysis, in accordance with national guidelines and DHS Program policies.

## Conflicts of Interest

The authors declare no conflicts of interest.

## Transparency Statement

Ololade Julius Baruwa affirms that this manuscript is an honest, accurate, and transparent account of the study being reported; that no important aspects of the study have been omitted; and that any discrepancies from the study as planned have been explained.

## Supporting information


Supporting File

## Data Availability

The study used secondary data from dhsprogram.org, which is publicly available at https://dhsprogram.com/data/available-datasets.cfm. O.J.B. had full access to all study data and takes responsibility for the integrity of the data and the accuracy of the data analysis.
