# Supplementary material for: Awareness of Abortion Legality and Safe Abortion Uptake in Ghana: Assessing the Moderating Role of Education in a Cross‐Sectional Study
Source: Health Sci Rep. 2026 Jul 30;9(8):e72928. doi: 10.1002/hsr2.72928 (PMC13420288; doi:10.1002/hsr2.72928)
Supplement: Supplementary file 1 — Supporting File [file HSR2-9-e72928-s001.docx]

**Supplementary Table 1: Variance inflation factor and tolerance in the multivariable regression analysis**

|  | **VIF** | **Tolerance** |
| --- | --- | --- |
| Variables |  |  |
| Parity | 2.17 | 0.460462 |
| Age | 1.70 | 0.586943 |
| Household wealth index | 1.49 | 0.671714 |
| Marital status | 1.47 | 0.680024 |
| Education | 1.32 | 0.760350 |
| Place of residence | 1.28 | 0.779912 |
| Ethnicity | 1.27 | 0.789357 |
| Reqion | 1.17 | 0.854089 |
| Religion | 1.08 | 0.924793 |
| Media exposure | 1.06 | 0.945660 |
| Reason for abortion | 1.05 | 0.951171 |
| Early sexual debut | 1.05 | 0.952910 |
| Legal bortion | 1.04 | 0.957105 |
| Contraceptive use | 1.02 | 0.980614 |
